# A Systems Biology and Drug Repositioning Approach for the Analysis of Mutual Genes Between Celiac Disease and Irritable Bowel Syndrome

**DOI:** 10.1155/bmri/8227229

**Published:** 2025-12-21

**Authors:** Haitham Al-Madhagi

**Affiliations:** ^1^ Biochemical Technology Program, Dhamar University, Dhamar, Yemen

**Keywords:** celiac disease, drug repurposing, gene enrichment, hub genes, irritable bowel syndrome, systems biology

## Abstract

Celiac disease (CD) and irritable bowel syndrome (IBS) are two disorders that share common features, such as similar symptoms and autoimmune involvement. However, the molecular genetic mechanisms underlying their pathogenesis remain unclear. An in silico systems biology approach was performed to analyze the RNA‐seq (GSE146190 and GSE166869) and microarray data (GSE164883 and GSE63379) of both diseases. Gene ontology was first identified, followed by transcriptional factors and miRNAs that regulate the mutual genes by Enrichr platform. Moreover, a protein–protein interaction network of the shared genes was constructed, and the hub genes were identified using Network Analyst and Cytoscape. Finally, the tertiary structure of the most significant hub gene product was downloaded and screened against approved drugs using DrupRep server for drug repurposing. Four hundred thirty‐nine shared genes between CD and IBS were revealed, which were mainly involved in response to stimulus, proliferation regulation, metabolism of small molecules, and apoptosis. RARG, NFE2L2, VDR, NCOA1, and RXRA were the top five transcription factors that regulated these genes, whereas hsa‐miR‐4632‐3p, hsa‐miR‐598‐5p, hsa‐miR‐7108‐3p, and hsa‐miR‐29b‐3p were the top five miRNAs. SRC, STAT1, CCNB1, CDK1, CD44, RRM2, ERBB2, BUB1B, KIF11, and TOP2A were ranked as the Top 10 hub genes by the PPI network analysis. Temoporfin, rimegepant, and eltrombopag were suggested as the top three lead candidates by the virtual screening against SRC with binding affinities of −11.1, 10.9, and −10.8 kcal/mol, respectively. These drugs are potential SRC inhibitors that warrant further experimental validation. Novel insights into the molecular genetic mechanisms of CD and IBS and new therapeutic avenues for these disorders were provided by this study.

## 1. Introduction

Celiac disease (CD) is an autoimmune disorder triggered by the consumption of gluten, a protein found in wheat and other grains. It occurs in genetically predisposed individuals, affecting approximately 1% of the global population [1]. Upon gluten consumption, the immune response damages the small intestine, leading to a variety of symptoms. While about 2.5% of young adults worldwide carry this genetic predisposition, only a fraction of them develops the full‐blown disease [2]. CD can manifest at any age, including in the elderly, and its clinical presentation is increasingly recognized as complex and diverse [3]. Improved serological tests and biopsy protocols have enhanced detection, contributing to a rising reported incidence of the condition worldwide [4].

Although the exact cause for rising CD rates remains elusive, environmental factors are strongly suspected, given the parallel increase in other autoimmune disorders [5]. Some theories attribute the surge to modern Western dietary habits, including increased gluten consumption, or to changes in wheat from agricultural practices. However, these claims remain unproven [6, 7].

Diagnosis typically involves detecting antibodies to transglutaminase 2 enzyme in the blood, which are highly specific to CD. The production of these antibodies is linked to how the immune system recognizes gluten, utilizing human leukocyte antigen (HLA‐DQ) molecules to present modified gluten peptides to immune cells, triggering the immune response and antibody production [8, 9].

Gluten‐rich sources like wheat, rye, and barley are known allergens, whereas oats, although gluten‐free, may still provoke reactions in some individuals [10]. Current guidelines recommend initially adopting a strict gluten‐free diet that excludes oats, with the option to reintroduce certified uncontaminated oats once the patient is stable [11]. However, due to the high risk of gluten cross‐contamination in commercial oat supplies, which is a particular concern in developing nations, many clinicians advise celiac patients to avoid oats entirely as a precaution [12]. The World Health Organization (WHO) has established standards for gluten‐free labeling in foods, stipulating a maximum gluten content of 20 parts per million (ppm) [13].

In fact, an increasing number of patients in the United States suffer gastrointestinal (GI) symptoms, such as abdominal pain (21.8 million) and diarrhea (3.4 million) is noticeable. Managing these symptoms can be costly and requires hospitalization and testing [14]. Irritable bowel syndrome (IBS) is a common GI disorder that causes abdominal pain and changes in stool [15]. It can be divided into three major types based on stool pattern: IBS‐C (with constipation, 28.5%), IBS‐D (with diarrhea, 35.0%), or IBS‐M (mixed, 31.0%) [15]. The US prevalence of IBS is estimated to be between 4.8% and 5.3%, which is higher than CD (0.7%) and IBD (0.5%) [16, 17]. The best way to diagnose and treat IBS depends on the main symptoms (e.g., pain, discomfort, and bloating) and the stool type (e.g., diarrhea, constipation, or mixed) [18]. Therefore, different IBS subtypes may need different approaches. IBS is a chronic condition that affects how the gut and brain communicate. It has no clear cause, but it involves many factors, such as changes in gut bacteria, sensitivity, movement, and immunity [19, 20].

Some people develop IBS‐D after having a gut infection [21, 22]. In Europe, IBS is essentially treated with musculotropic spasmolytic agents like otilonium bromide (OB). In tertiary care, a low fermentable oligosaccharides, disaccharides, monosaccharides, and polyols (FODMAP) diet provides significant improvement. Notably, the latter option was demonstrated by randomized clinical trials to be superior to OB and should, therefore, be considered as the first‐treatment option for IBS in the primary care context [23].

Indeed, CD and IBS share numerous clinical and pathophysiological features, including immune system involvement, intestinal manifestations, overlapping symptoms, and gut dysbiosis. Analysis of urinary volatile organic compounds (VOCs), which are byproducts of microbial fermentation, shows promise as a tool to distinguish between the two conditions [24]. However, this symptomatic overlap often makes differential diagnosis challenging. Furthermore, current gastroenterological guidelines recommend similar dietary therapies, namely, a gluten‐free diet or a low‐FODMAP diet, for managing symptoms in both CD and IBS [25].

Recent research has unraveled several new druggable targets and investigational drugs for both conditions, reflecting a truer understanding of the underlying mechanisms. Serotonin receptors, including 5‐HT3 and 5‐HT4, guanylate cyclase‐C, and several purinergic receptors including P2X3 and P2Y, are potential targets under investigation as gut motility or visceral pain modulators for the treatment of IBS [26]. Clinical trials suggest that investigational drugs including ibodutant (a tachykinin NK2 receptor antagonist) and solabegron (a *β*3‐adrenergic receptor agonist) provide superior symptom relief for IBS‐D patients compared with existing therapies. There is also interest in cannabinoid‐based treatments and treatments targeting the microbiome, with both strategies offering promise for symptom improvement in IBS due to the modulation of gut flora and inflammation [27]. New approaches for the treatment of CD rely on the inhibition of the transglutaminase enzyme involved in gluten modification or use enzyme therapies, including those that degrade gluten before it can provoke an immune response. Another approach is the development of immunotherapies that desensitize the immune system to gluten and hence offer hope for patients with very strict gluten‐free diets [28]. These developments point toward a more specific approach to such GI diseases by targeting the particular pathophysiological processes involved. However, targeting traditional targets with newer drug entities will only reduce side effects without fully treating the condition. Also, the long‐term benefits and side effects of these therapies are yet to be fully understood, and the race for novel druggable targets and repurposed drugs is ongoing.

While CD and IBS are distinct disorders, they present with similar GI symptoms, often complicating diagnosis. To our knowledge, a comparative transcriptomic analysis to identify shared molecular signatures between CD and IBS has not been conducted. This gap limits our understanding of their potential pathophysiological overlap and hinders the development of novel therapeutic strategies. Therefore, this study was designed to (1) identify mutual differentially expressed genes (DEGs) in CD and IBS; (2) predict the most central hub genes within these shared pathways; and (3) perform computational drug repurposing for the top‐ranked hub gene. The ultimate goal is to propose a repurposed drug candidate that could serve as a novel therapeutic option for symptoms common to both conditions.

## 2. Materials and Methods

### 2.1. Data Extraction and Processing

Gene expression omnibus (GEO) repository [29] (https://www.ncbi.nlm.nih.gov/geo/) was used a source for retrieving the raw data having the accession code GSE146190 (duodenal biopsies of five healthy controls and 11 histology proven CD), GSE164883 (25 CD duodenal probes and 21 control samples) for CD and GSE166869 (26 IBS patients and 15 healthy volunteers), and GSE63379 (35 IBS samples and 32 healthy control) for IBS (accessed on October 9, 2025). While GSE146190 and GSE166869 were RNA‐seq data, GSE164883 and GSE63379 were microarray datasets. The transcriptome of two types of data was processed with GEO2R embedded tool [30] (https://www.ncbi.nlm.nih.gov/geo/geo2r/) which can be used for the comparison of two or more transcriptomic samples to identify DEGs with the assistance of GEO query and limma R packages. The shortlisting is based upon log2FC < −1 for sorting down‐regulated genes and log2FC > 1 for up‐regulated genes with a *p* value < 0.05. DEGs of the two data sets are then submitted to Venny 2.1 online tool (https://bioinfogp.cnb.csic.es/tools/venny/) for drawing and concluding the mutual genes.

### 2.2. Gene Enrichment and Pathway Analysis

To give an overall overview of the mutual gene roles, the gprofiler platform [31] (https://biit.cs.ut.ee/gprofiler/) was employed. This involved biological processes, cellular compartments, molecular function, as well as the Kyoto Encyclopedia of Genes and Genomes (KEGG). An adjusted *p* value less than 0.05 was a cut off criterion for statistical significance.

### 2.3. Identification of Transcriptional and Posttranscriptional Regulators

The mutual genes were also investigated for the identification of transcriptional regulation (via transcriptional factors, TF) and posttranscriptional (via miRNA). This was accomplished by utilizing the Enrichr server [32] (https://maayanlab.cloud/Enrichr/). The TF network was predicted according to the TRANSFAC database, whereas miRNAs were based on the miRTarBase database. The top 10 of the two regulators were tabulated according to *p*‐value.

### 2.4. Hub Gene Network Analysis

The mutual genes were then submitted to NetworkAnalyst [33] (https://www.networkanalyst.ca/) to infer the protein–protein interactions (PPIs)via STRING Interactome dataset with a confidence score cutoff of 700. Additionally, to identify the hub genes, we first selected the mutual genes that had potential PPIs using STRING‐db [34] (https://string-db.org/). Then, we filtered the PPIs by a combined score of more than 0.7 to increase the reliability of the analysis. This was followed by importing the PPI network into Cytoscape v.3.9.1 [35] and using the CytoHubba plugin to find the 10 most important genes based on the degree of connections. Degree filtration means to sort the genes with the highest number of connections with other genes, reflecting the degree of significance and implication of this gene in the pathology in question.

### 2.5. Hub Gene‐Drug Interactions

The potential hub genes–drugs interaction was explored via NetworkAnalyst [33] (https://www.networkanalyst.ca/). Only the top‐ranked hub gene and the food and drug authority (FDA)‐approved drugs were involved in the prediction. This will ensure the list of available approved drugs that can be used as blockers of the hub gene.

### 2.6. Receptor Preprocessing

From the Protein Data Bank (PDB), the tertiary architecture of the SRC, the most significant hub protein, was downloaded via the accession ID of 2SRC. The 3D structure was discovered by X‐ray crystallography with a resolution of 1.50 Å. The receptor is a single chain with 452 amino acids [36]. All non‐protein atoms, including the complexed synthetic inhibitor, were eliminated from the PDB file via UCSF Chimera v. One.16 program [37] and processed by the Dockprep module. This step is mandatory prior to molecular docking, as these compounds would otherwise compete with the drug library for binding at the same active site, leading to inappropriate findings.

### 2.7. Active Site Prediction

The molecular docking must be preceded by the identification of the pocket of the protein to which the docking process will be conducted. The active pocket was predicted through the PrankWEB 3 tool (https://prankweb.cz/) [38]. The server prioritizes pockets according to combined parameters such as conservation, amino acid residues in every pocket, probability score, and then ranks the pockets accordingly.

### 2.8. Virtual Screening

The last step was to virtually repurpose FDA‐approved drugs against the SRC. This was conducted by the DrugRep server (http://cao.labshare.cn:10180/DrugRep/) [39], selecting pocket 5 (center values: 19, 25, 57 and size coordinates: 17, 23, 16 with exhaustiveness of 8) that is compatible with the active site prediction deployed earlier by using PrankWEB 3. The FDA drug list is a pre‐existing library retrieved from DrugBank. In addition, the server also provides a table for Lipinski′s rule of five (Ro5) for the best hits.

## 3. Results

### 3.1. Data Extraction and Processing

The first step was to ensure the normalization of the DEGs of the two datasets. This was confirmed by the volcano plot, which shows the distribution of all genes in addition to the DEGs as provided in Figure 1. The under‐expressed genes are shown in blue, whereas the over‐expressed genes are shown in red. The analyzed gene expression data displayed about 4669 DEGs from the CD data, whereas the DEGs in the IBS were found to be 1234 DEGs. In mutual, 439 genes were found in common as depicted in Figure 1c. Such large numbers signify the similarities between the two diseases, as both are autoimmune disorders and occur in the intestine of mankind. Therefore, only the mutual genes were selected for further exploration.

Figure 1Volcano plots of RNA‐seq and microarray data of CD (a) and IBS (b) as computed using the GEO2R tool. The mutual and DEGs between CD and IBS are also shown (c).

**Figure:**
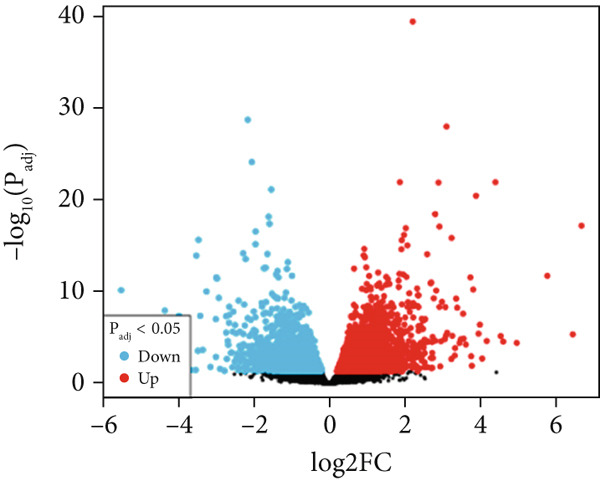
(a)

**Figure:**
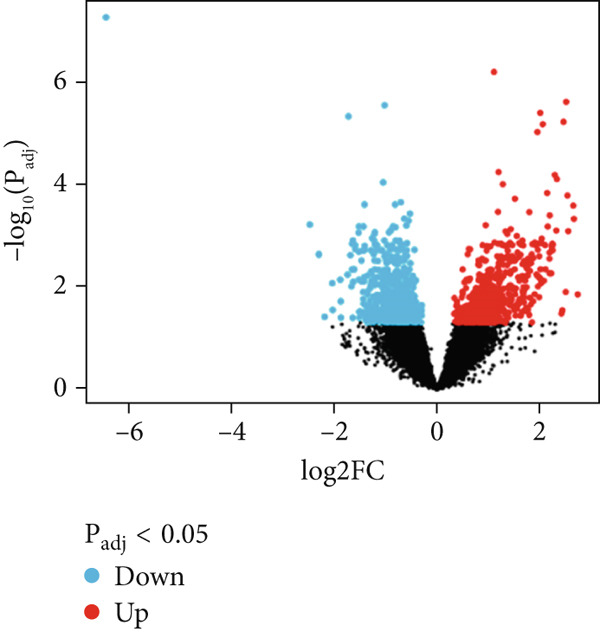
(b)

**Figure:**
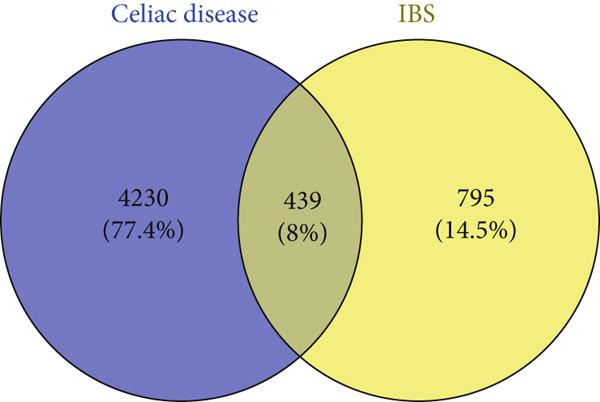
(c)

### 3.2. Gene Enrichment and Pathway Analysis

The DEGs were enriched to discover the corresponding cellular compartments (CC), biological processes (BP), and the molecular functions (MF). It turned out that vesicle, cell periphery, brush border membrane, and the endomembrane system were the top CC of the DEGs, indicative of the roles played by these genes at the membrane or outside the enterocytes. Concerning the biological processes, DEGs share in mutual processes such as response to stimulus, proliferation regulation, metabolism of small molecules, and apoptosis. The MF stimulus response is compatible with the output of the CC mentioned. The gene enrichment findings of the DEGs are summarized in Figure 2.

Figure 2Gene enrichment overview (a) and detailed CC, BP, and MF of the enriched genes. (b) Each gene ontology finding was ranked for the enriched genes according to the adjusted *p* value (P_adj_) along with the negative logarithmic P_adj_ (‐log_10_ P_adj_).

**Figure:**
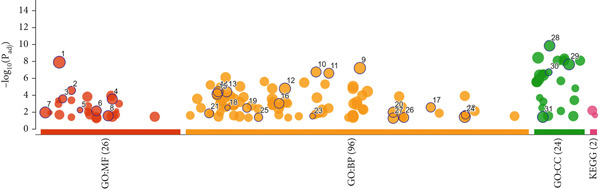
(a)

**Figure:**
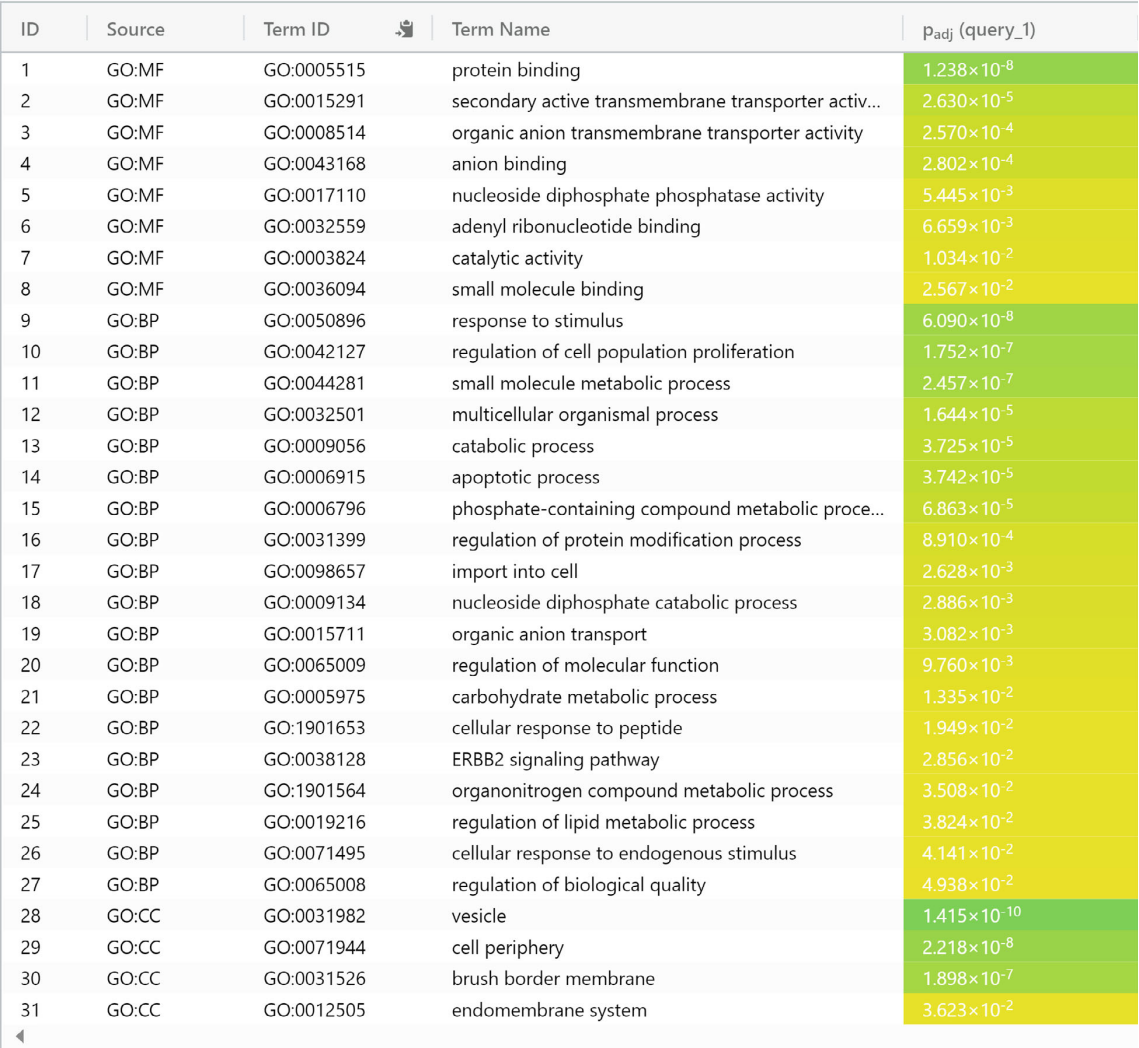
(b)

### 3.3. Prediction of PPI Network

**Figure 3:**
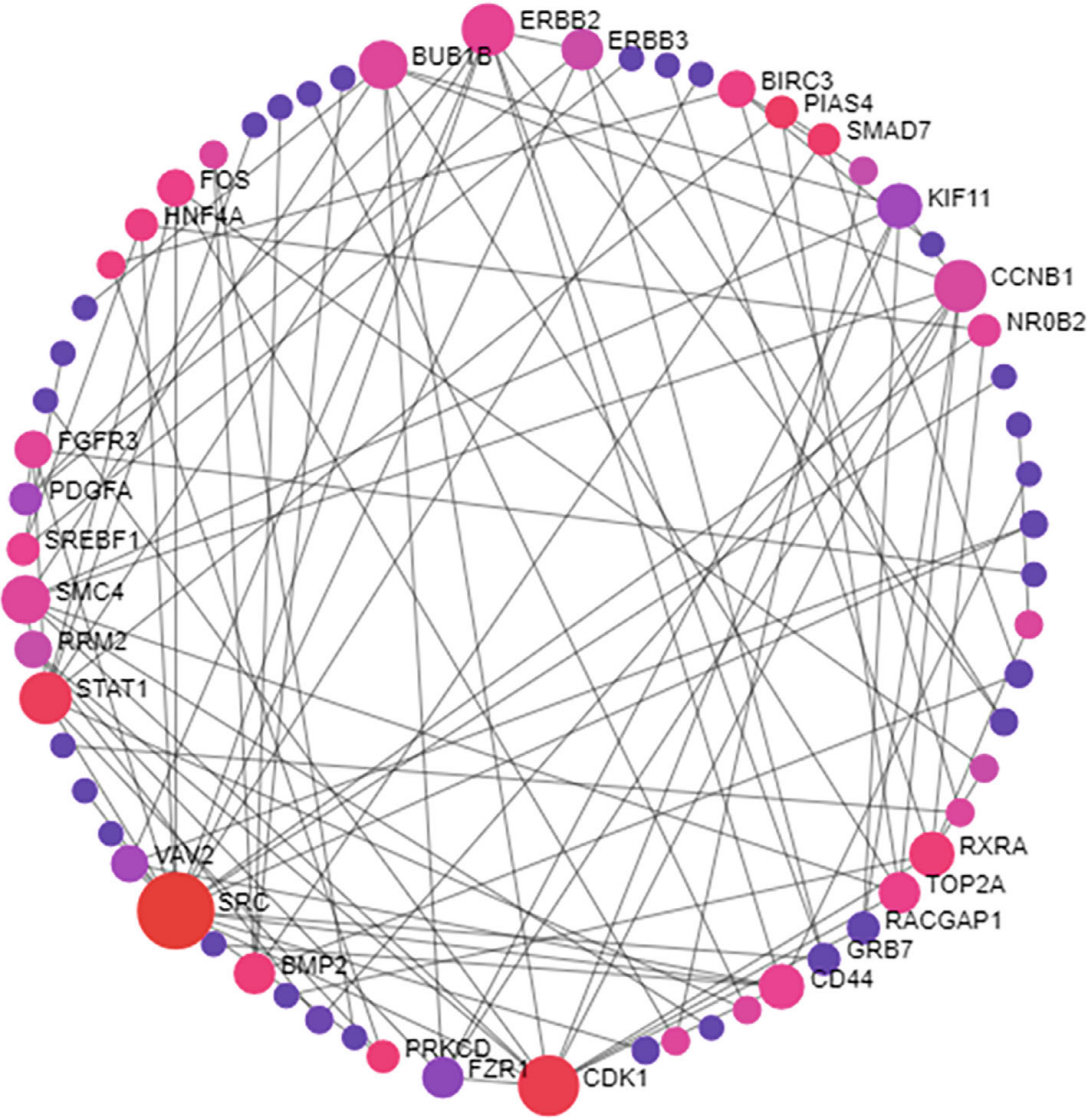
PPI of the mutual genes between CD and IBS as generated by NetworkAnalyst. Figure provides the minimum network with the highest nodes connections with the higher the circle is the more interacting the gene is. Also, the degree of gene connection is colored from magenta (low) to red (high).

According to STRING analysis, the number of nodes of the mutual genes were 398, edges 259, with an average node degree of 1.3 and PPI enrichment *p* value of < 1.0e‐16. This reflects the high degree of connections between the shared genes and the high confidence of this prediction. The gene network is illustrated in Figure 3. From Figure 4, as the gene circle enlarges, high degree of connections is considered. Similarly, the degree of connections is also monitored by the change in color from violet (low‐connections) to pink (high‐connections). Accordingly, SRC, CDK1, and STAT1 were the top three genes with highest node connections but this make it difficult to infer the hub genes which necessitate the extraction of hub genes from this network through deploying the Cytoscape program.

Figure 4Top 10 hub genes of the shared genes (a) and their corresponding ranking (b). Prioritization was performed based on the degree of gene interaction with other genes, giving rise to SRC as the most interacting (hub) gene.

**Figure:**
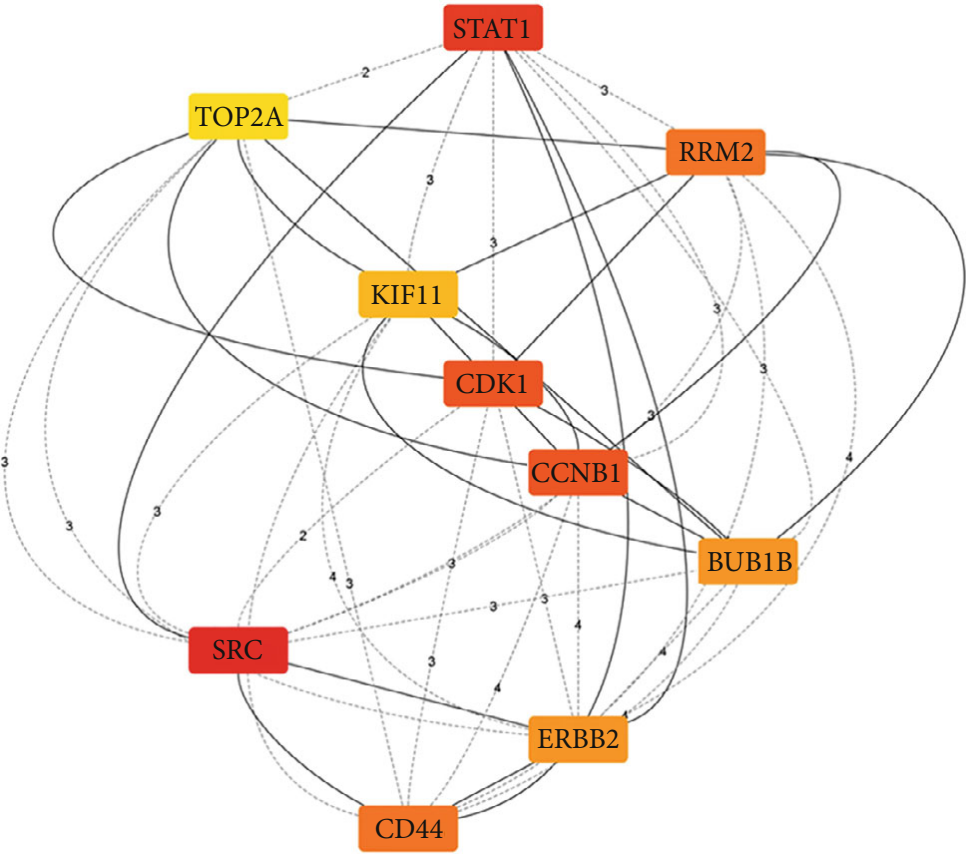
(a)

**Figure:**
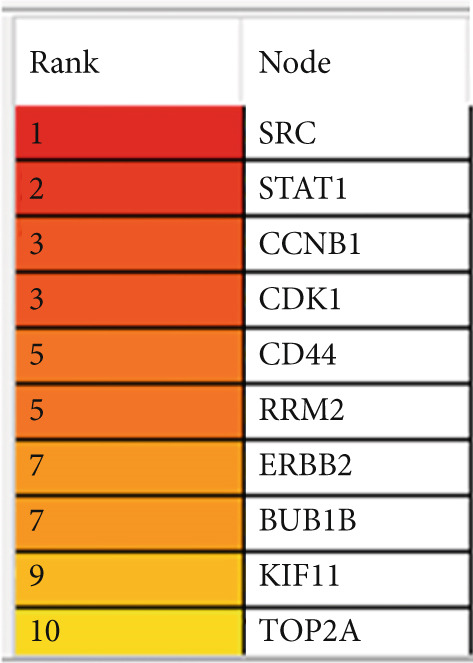
(b)

### 3.4. Prediction of Hub Genes

To infer the hub genes of the mutual genes between CD and IBS, the Cytohubba plugin of the Cytoscape program was utilized. The hub genes were extracted based on the degree of node connections. The 10 hub genes of shared genes between CD and IBS are ranked descendingly as SRC, STAT1, CCNB1, CDK1, CD44, RRM2, ERBB2, BUB1B, KIF11, and TOP2A as displayed in Figure 4.

### 3.5. Prediction of Regulating TFs and miRNAs

The expressed genes are subjected to control at the transcription level as well as posttranscriptional process; this is achieved by TFs and miRNAs, respectively. To decipher the potential TFs, the Enrichr platform was employed. The platform ranked RARG, NFE2L2, VDR, NCOA1, RXRA, STAT1, FOXM1, HDAC2, ESRRA, and CTNNB1 according to the significance level (Table 1).

**Table 1:** Top‐10 most important transcriptional factors regulating the mutual genes.

**Index**	**Name**	**p**
1	RARG	0.001622
2	NFE2L2	0.001998
3	VDR	0.002525
4	NCOA1	0.003147
5	RXRA	0.003267
6	STAT1	0.003672
7	FOXM1	0.004257
8	HDAC2	0.004713
9	ESRRA	0.004772
10	CTNNB1	0.006191

These TFs are predicted with high confidence as reflected by the highly statistical significance levels (*p* < 0.01). On the other hand, hsa‐miR‐4632‐3p, mmu‐miR‐337‐3p, hsa‐miR‐598‐5p, hsa‐miR‐7108‐3p, hsa‐miR‐29b‐3p, hsa‐miR‐4707‐3p, hsa‐miR‐29a‐3p, hsa‐miR‐26b‐5p, and hsa‐miR‐301a‐5pwere the top‐ranked miRNAs that regulate the shared genes posttranscriptionally (Table 2). The coverage of control of the corresponding genes by TFs and miRNAs are provided in Figure 5.

**Table 2:** The most significant miRNAs controlling the shared genes.

**Index**	**Name**	**p**
1	hsa‐miR‐4632‐3p	0.0009972
3	hsa‐miR‐598‐5p	0.001451
4	hsa‐miR‐7108‐3p	0.001476
5	hsa‐miR‐29b‐3p	0.001707
6	Hsa‐miR‐4707‐3p	0.001878
7	hsa‐miR‐29a‐3p	0.001898
8	hsa‐miR‐26b‐5p	0.003263
9	hsa‐miR‐301a‐5p	0.003472

Figure 5The regulation coverage of the TF (a) and miRNA (b) on the most important 20 genes.

**Figure:**
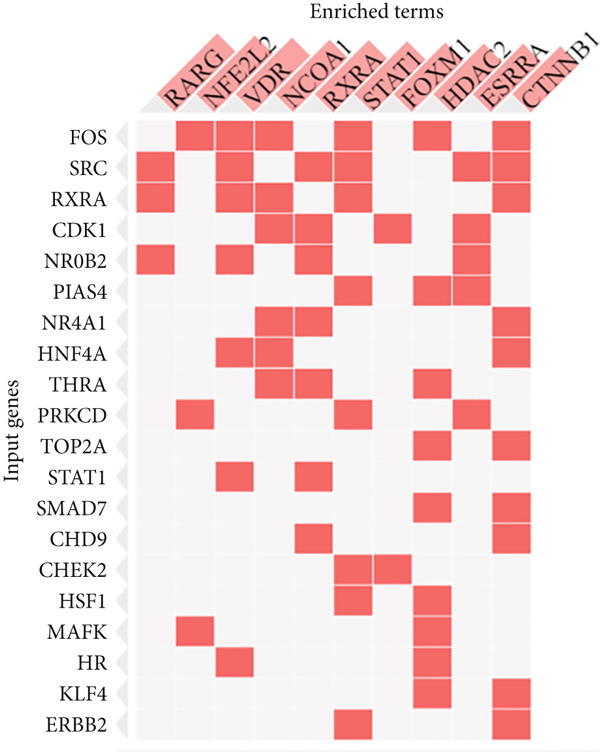
(a)

**Figure:**
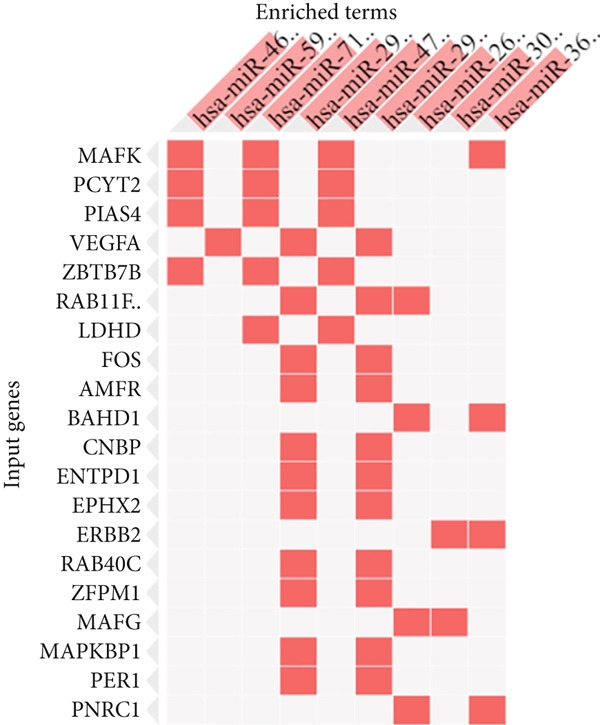
(b)

### 3.6. Hub Gene‐Drug Interaction

The interaction between the top hub gene SRC and the approved drugs has been made available via searching the Drugbank database. Results showed that six drugs, namely, dasatinib, tirbanibulin, bosutinib, ponatinib, nintedanib, and fostamatinib were found to be specific drugs against the SRC target and already gained Food and Drug Authority (FDA) approval. It should be noted that all the listed drugs are pharmacologically classified as inhibitors of SRC activity and, hence, are hands‐on therapeutic options. The SRC drugs are listed in Table 3.

**Table 3:** The available FDA‐approved drugs against the most significant hub gene SRC.

**Hub gene**	**Drug**	**Drug Bank ID**	**Status**
SRC	Dasatinib	DB01254	Approved
	Tirbanibulin	DB06137	Approved
	Bosutinib	DB06616	Approved
	Ponatinib	DB08901	Approved, investigational
	Nintedanib	DB09079	Approved
	Fostamatinib	DB12010	Approved, investigational

### 3.7. Active Site Prediction

To ensure that the molecular docking is taking place right at the active center of the SRC enzyme, the active site was predicted using PrankWeb 3 server. The server listed 4 probable pockets that would serve as the active site of the enzyme. These pockets were listed in Table 4 and their corresponding positions in the enzyme structure are provided in Figure 6. Indeed, pocket 1 was the best potential one in terms of score (53.37), probability (0.981), and conservation (2.163) and, thus, ranked first as the most potential active site of the SRC enzyme. Accordingly, the molecular docking was carried out in this pocket, which is greatly compatible with pocket 5 in the DrugRep server, the tool deployed for virtual screening.

**Table 4:** The predicted active pockets of the SRC enzyme and their corresponding properties.

**Pocket**	**Rank**	**Score**	**Probability**	**Conservation**	**Amino acid Residue ID**
Pocket 1	1	53.37	0.981	2.163	A_273 A_274 A_275 A_276 A_277 A_278 A_279 A_281 A_293 A_295 A_297 A_302 A_307 A_314 A_323 A_325 A_336 A_338 A_340 A_341 A_342 A_344 A_345 A_348 A_386 A_388 A_390 A_391 A_393 A_403 A_404 A_405 A_407 A_408 A_411 A_416 A_423 A_425
Pocket 2	2	2.07	0.045	0.496	A_136 A_249 A_250 A_251 A_252 A_290 A_291 A_326 A_339 A_340 A_91 A_92 A_93 A_95
Pocket 3	3	1.78	0.032	1.124	A_154 A_318 A_319 A_320 A_372 A_376 A_379 A_513
Pocket 4	4	1.63	0.026	NP	A_135 A_144 A_146 A_147 A_149 A_150 A_247 A_248 A_89 A_90

Abbreviation: NP: not provided.

Figure 6The position of the predicted pockets (a) along with the best pocket alone (b) of the hub protein SRC.

**Figure:**
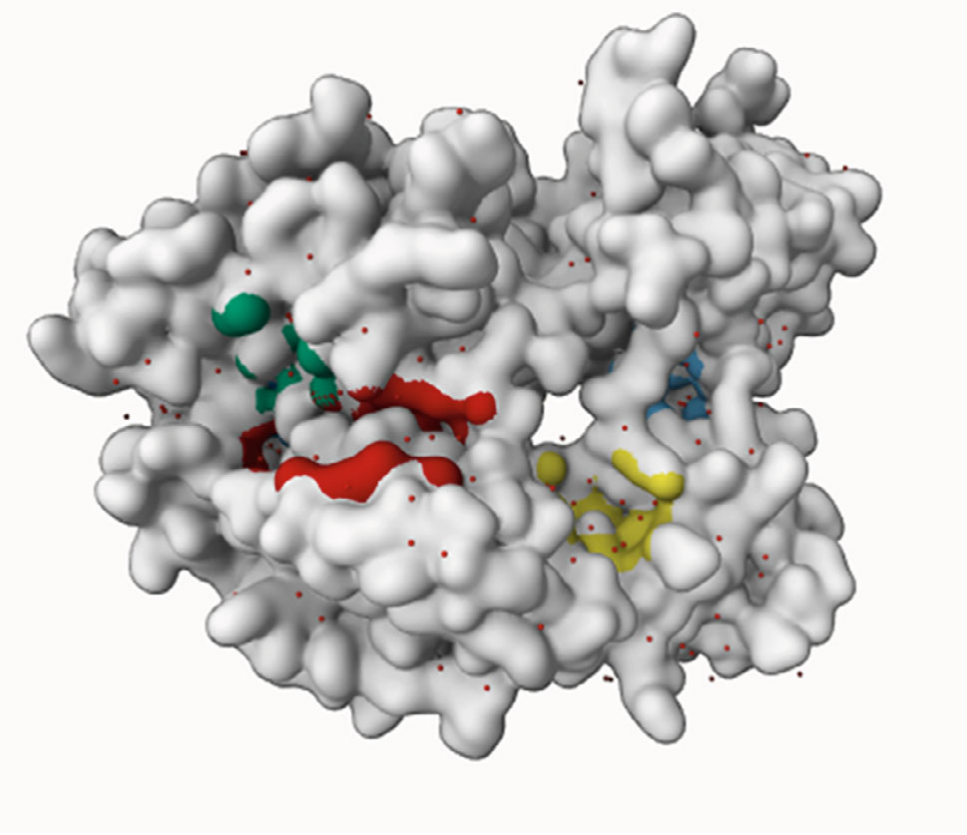
(a)

**Figure:**
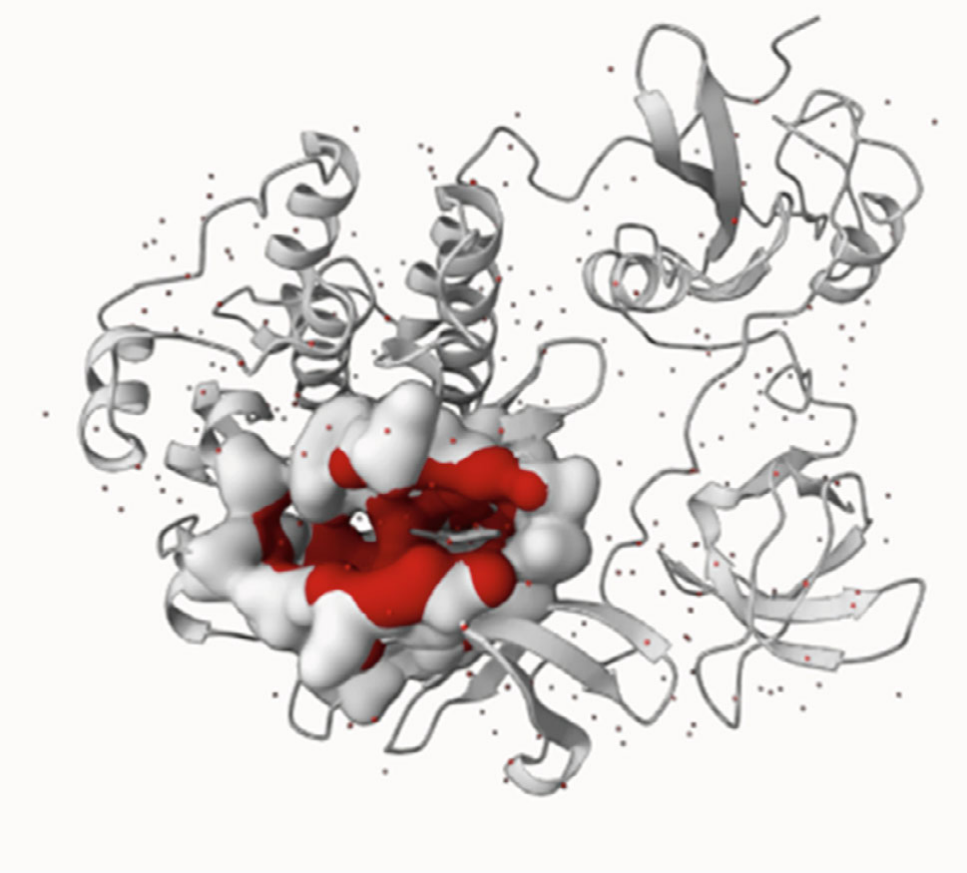
(b)

### 3.8. Virtual Screening

**Figure 7:**
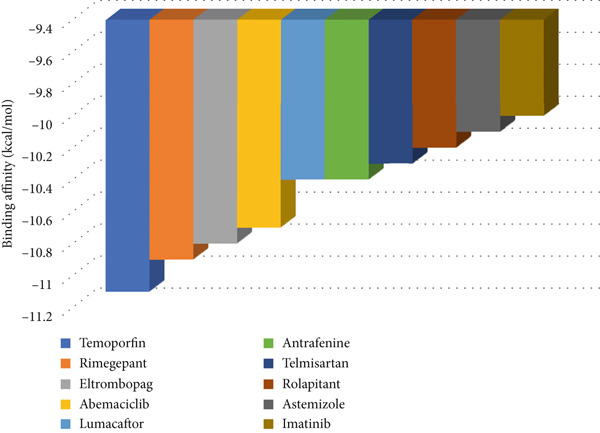
Binding affinity of the Top 10 FDA‐approved drugs against SRC as provided by DrugRep.

The virtual screening results of the DrugRep server identified several FDA‐approved drugs that can be repurposed as inhibitors to SRC. The finding of the virtual repurposing is summarized in Figure 7. This includes temoporfin, rimegepant, and eltrombopag, the top three lead candidates, with binding affinities of −11.1, 10.9, and −10.8 kcal/mol, respectively. The other drug‐likeness properties of the top 10 candidates are also listed in Table 5. Some of them exhibited violations of the Ro5 such as having molecular weight > 500 or LogP > 5. However, this cannot be deemed a medicinal issue given that they all are already gained the approval of FDA.

**Table 5:** Ro5 of the Top 10 repurposed FDA‐approved drugs against SRC ranked by docking scores.

**Name**	**Formula**	**MW**	**HBD**	**HBA**	**RB**	**Rings**	**LogP**
Temoporfin	C_44_H_32_N_4_O_4_	680.764	4	6	8	9	8.7
Rimegepant	C_28_H_28_F_2_N_6_O_3_	534.568	1	4	5	6	3.5
Eltrombopag	C_25_H_22_N_4_O_4_	442.4666	3	4	7	4	4.7
Abemaciclib	C_27_H_32_F_2_N_8_	506.606	1	4	7	5	3.8
Lumacaftor	C_24_H_18_F_2_N_2_O_5_	452.414	2	4	7	5	4.4
Antrafenine	C_30_H_26_F_6_N_4_O_2_	588.5435	1	2	8	5	7.5
Telmisartan	C_33_H_30_N_4_O_2_	514.6169	1	4	8	6	6.9
Rolapitant	C_25_H_26_F6N_2_O_2_	500.485	2	1	5	4	4.4
Astemizole	C_28_H_31_FN_4_O	458.5703	1	1	8	5	5.9
Imatinib	C_29_H_31_N_7_O	493.6027	2	4	8	5	3.5

Abbreviation: HBA: hydrogen bond acceptor; HBD: hydrogen bond donor; MW: molecular weight; RB: rotatable bonds.

**Figure 8:**
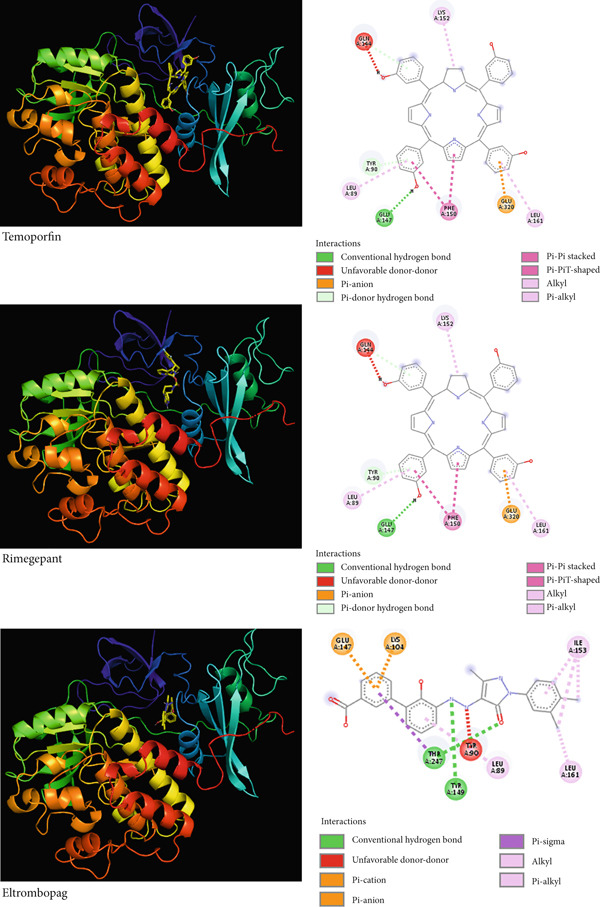
3D (left) and 2D (right) protein–ligand interactions of the top three drug candidates against SRC showing its fitness to the active site and the type of interactions.

The pattern of interactions formed between the top three drug candidates, temoporfin, rimegepant, and eltrombopag to the active site of SRC enzyme unveiled different findings. They displayed H‐bonds, many hydrophobic interactions in addition to some unfavorable interactions (similar charges approximation) as summarized in Figure 8.

## 4. Discussion

A significant number of individuals are not aware of the coexistence of CD and IBS. It is crucial to recognize that individuals diagnosed with CD who adhere to a strict gluten‐free diet may still experience symptoms, which could potentially be attributed to the presence of IBS. This is supported by studies showing a significantly higher prevalence of positive CD serology and biopsy‐proven CD in subjects with IBS‐like symptoms compared with healthy controls [40, 41]. Therefore, the current study was designed to identify shared genes between CD and IBS and to provide a comprehensive bioinformatics analysis to decipher hub genes for targeting with a library of repurposed FDA‐approved drugs.

A comparison of the DEGs from CD and IBS yielded 439 shared genes. The gene enrichment unveiled the shared genes affect the membrane and extracellular regions of the enterocytes, as well as their functions in metabolism, proliferation, apoptosis, and stimulus response. These changes reflect the altered cellular states and responses of the enterocytes in CD and IBS, which are exposed to different inflammatory signals in the small intestine. The MF stimulus response is compatible with the output of the CC mentioned. This could be interpreted by the fact that CD, as well as IBS, are exposed regularly to pro‐inflammatory and anti‐inflammatory signals in the small intestine niche.

Moreover, STRING analysis of the mutual genes revealed a network of 398 nodes and 259 edges, with an average node degree of 1.3 and a PPI enrichment *p* value of < 1.0e−16. These results signify a high degree of connectivity among the shared genes and high confidence in the predicted interactions. This network was then utilized to discover the most significant hub genes (Top 10). SRC, STAT1, CCNB1, CDK1, CD44, RRM2, ERBB2, BUB1B, KIF11, and TOP2A were the most important genes (hub genes) with highest node connections as computed by CytoHubba pluggin of Cytoscape software. These genes were found to be regulated by RARG, NFE2L2, VDR, NCOA1, RXRA, STAT1, FOXM1, HDAC2, ESRRA, and CTNNB1 at the transcriptional level (under TF regulation) and also by hsa‐miR‐4632‐3p, mmu‐miR‐337‐3p, hsa‐miR‐598‐5p, hsa‐miR‐7108‐3p, hsa‐miR‐29b‐3p, hsa‐miR‐4707‐3p, hsa‐miR‐29a‐3p, hsa‐miR‐26b‐5p, and hsa‐miR‐301a‐5p at the posttranscriptional level to block their translation (under miRNA control).

SRC is a proto‐oncogene that encodes a nonreceptor tyrosine kinase that regulates cell growth, differentiation, migration, and survival. SRC is activated by various growth factors and cytokines and mediates downstream signaling pathways, such as PI3K/AKT, MAPK/ERK, and JAK/STAT. SRC is overexpressed or mutated in many cancers and contributes to tumor initiation, invasion, angiogenesis, and metastasis. SRC inhibitors have been developed and tested in clinical trials for the treatment of solid tumors [42, 43]. This accounts for being the most significant hub genes enriched, shared between CD and IBS.

STAT1 is a transcription factor that mediates the cellular responses to interferons and other cytokines. STAT1 is activated by phosphorylation at tyrosine 701 by JAK kinases and translocates to the nucleus to regulate the expression of genes involved in immune response, apoptosis, cell cycle arrest, and DNA repair. STAT1 acts as a tumor suppressor or promoter depending on the cellular context and the type of cancer. STAT1 deficiency or mutation impairs the antitumor immune response and confers resistance to interferon therapy. STAT1 activation by oncogenic signaling or viral infection promotes tumor growth, survival, and angiogenesis. STAT1 inhibitors have been explored as potential anticancer agents [44].

CCNB1, a gene responsible for encoding cyclin B1, serves as a regulatory component within the cyclin‐dependent kinase 1 (CDK1) complex, which governs the progression from the G2 phase to the M phase of the cell cycle. The presence of CCNB1 is crucial for initiating mitotic entry, facilitating the assembly of the spindle apparatus, ensuring proper segregation of chromosomes, and promoting cytokinesis. Notably, CCNB1 is frequently observed to be overexpressed or amplified in various types of cancer, and this aberrant expression is often associated with unfavorable prognoses and resistance to chemotherapy. Consequently, researchers have developed CCNB1 inhibitors as a means to induce cell cycle arrest and trigger apoptosis specifically in cancer cells [45].

CDK1, formally known as cyclin‐dependent kinase 1, is a gene responsible for encoding a serine/threonine kinase that plays a pivotal role in regulating the transition from the G2 phase to the M phase of the cell cycle. Interestingly, CDK1 forms a complex with CCNB1 and is responsible for phosphorylating a range of substrates involved in mitosis, including nuclear lamins, histones, microtubule‐associated proteins, and checkpoint proteins. In many types of cancer, CDK1 is frequently observed to be overexpressed or activated, leading to enhanced tumor proliferation, invasion, and metastasis. To address this, researchers have developed CDK1 inhibitors that specifically target the CDK1/cyclin B1 complex or its ATP‐binding site. These inhibitors serve to induce cell cycle arrest and trigger apoptosis specifically in cancer cells [46, 47].

On the other hand, CD44 is a gene expressing a novel transmembrane glycoprotein that serves as a receptor for hyaluronic acid (HA) and other extracellular matrix components. CD44 mediates cell–cell and cell–matrix interactions, cell adhesion, migration, and invasion. CD44 also regulates intracellular signaling pathways, such as PI3K/AKT, MAPK/ERK, and NF‐*κ*B. CD44 is expressed in various normal tissues and cells, but its expression is altered in many cancers. CD44 is associated with tumor initiation, progression, metastasis, and drug resistance. CD44 also serves as a marker for cancer stem cells (CSCs), which are responsible for tumor heterogeneity, recurrence, and therapy failure. CD44 inhibitors have been developed to block the binding of CD44 to HA or its downstream signaling pathways and inhibit tumor growth, invasion, and metastasis [48, 49].

RRM2, a gene responsible for encoding ribonucleotide reductase subunit M2 (RRM2), plays a vital role in the conversion of ribonucleotides to deoxyribonucleotides (dNTPs). These dNTPs are crucial for DNA synthesis and repair processes. RRM2 expression is tightly regulated throughout the cell cycle, with high levels observed during the S phase. In numerous cancer types, RRM2 is frequently overexpressed or amplified, providing support for tumor proliferation, survival, and genomic instability. To counteract these effects, researchers have developed RRM2 inhibitors. These inhibitors work by depleting the dNTP pool, ultimately inducing DNA damage and triggering apoptosis specifically in cancer cells [50, 51].

ERBB2 is a member of the EGFR family of receptor tyrosine kinases that regulates cell signaling pathways related to cell growth, survival, invasion, and metastasis. ERBB2 can pair with itself or other EGFR family members and trigger the activation of PI3K/AKT, MAPK/ERK, and JAK/STAT cascades. ERBB2 is often overexpressed or amplified in various cancers, particularly breast cancer, and enhances tumor aggressiveness and malignancy. ERBB2 inhibitors have been designed to interfere with the extracellular domain, the intracellular kinase domain, or the dimerization interface of ERBB2 and block its signaling and function [52, 53].

BUB1B is one of the serine/threonine kinases that perform a pivotal role in the spindle assembly checkpoint (SAC), which ensures the proper alignment and segregation of chromosomes during mitosis. BUB1B phosphorylates various substrates involved in the SAC, such as BUB3, MAD1, MAD2, and CDC20. BUB1B is overexpressed or mutated in many cancers and contributes to chromosomal instability, aneuploidy, and tumorigenesis. BUB1B inhibitors have been developed to disrupt the SAC and induce mitotic arrest and apoptosis in cancer cells [54, 55].

KIF11 is a gene that encodes kinesin family member 11 (KIF11), a microtubule‐dependent motor protein that mediates the formation and function of the mitotic spindle. KIF11 is essential for chromosome alignment, segregation, and cytokinesis during mitosis. KIF11 is overexpressed or amplified in many cancers and correlates with poor prognosis and resistance to microtubule‐targeting agents. KIF11 inhibitors have been developed to interfere with the binding of KIF11 to microtubules or its ATPase activity and induce mitotic arrest and apoptosis in cancer cells [56, 57].

DNA topoisomerase II alpha is transcribed from TOP2A, which can change the shape of DNA by making temporary cuts in both strands. TOP2A participates in many processes that involve DNA, such as copying, reading, rearranging, and fixing. TOP2A is also a target for many drugs that fight cancer, such as anthracyclines, epipodophyllotoxins, and bisdioxopiperazines. These drugs bind to the complex formed by TOP2A and the cut DNA and prevent them from rejoining, causing DNA damage and cell death in cancer cells. TOP2A is often overexpressed or amplified in many cancers and indicates how will the cancer cells respond to the drugs that target TOP2A. TOP2A inhibitors have been created to increase the effectiveness of these drugs or to directly stop the enzyme from working [58].

**Figure 9:**
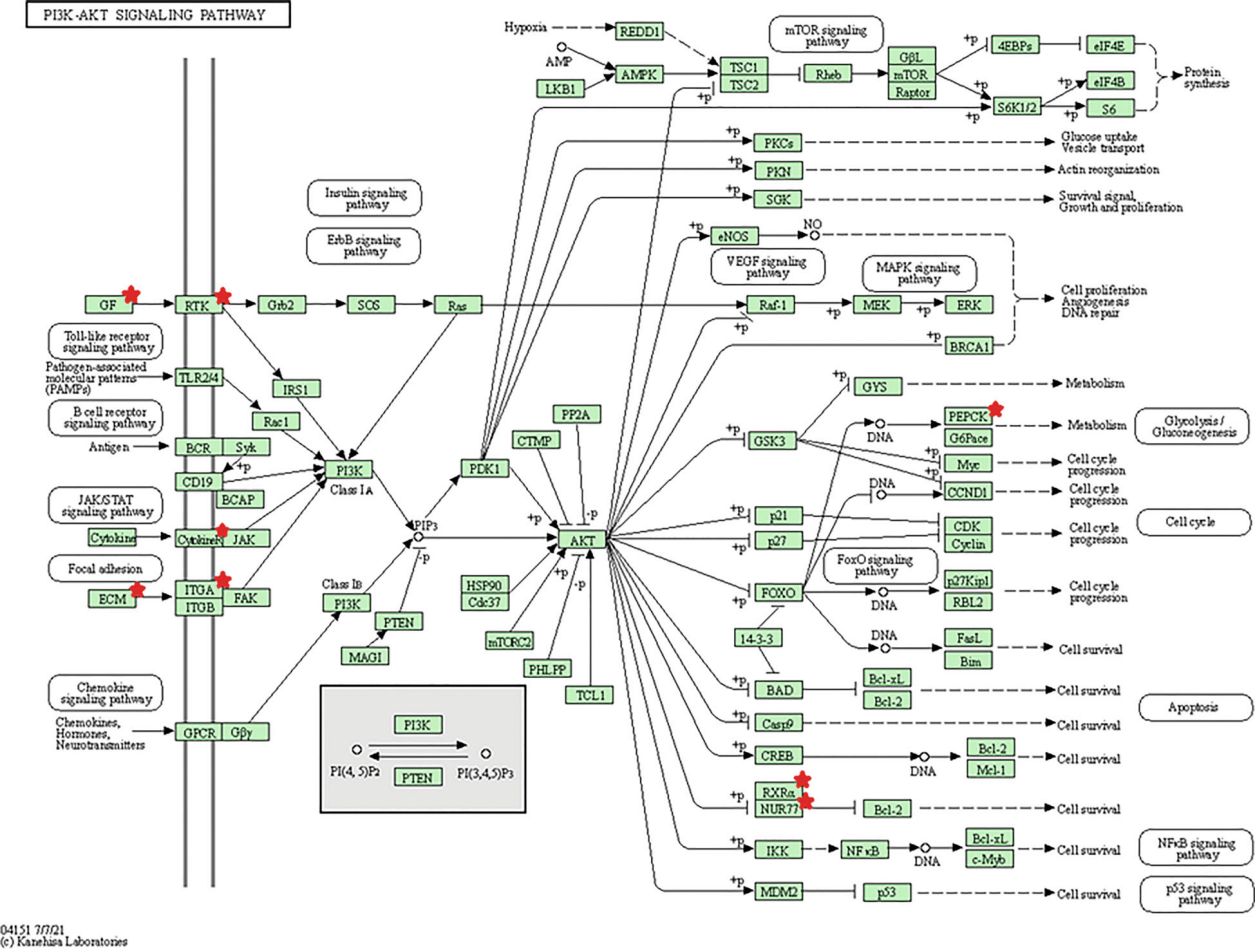
KEGG analysis of the second most enriched pathway, PI3K‐Akt signaling pathway. The implicated mutual genes are indiated in red asterisk.

Collectively, SRC, STAT1, CCNB1, CDK1, CD44, RRM2, ERBB2, BUB1B, KIF11, and TOP2A are genes that encode proteins involved in various cellular processes, such as signal transduction, cell cycle regulation, cell adhesion, DNA replication and repair, and mitosis. The question is what is the link between these cancer‐related genes and CD and IBS? The link word is “inflammation.” In reality, chronic inflammation is a risk factor for colorectal cancer in IBS. The risk depends on the duration, extent, and type of inflammation. Inflammation can cause changes in molecular pathways that are also involved in sporadic colorectal cancer, such as chromosomal instability, microsatellite instability, and hypermethylation. These changes can result from oxidative stress and the interaction of reactive molecules with genes related to cancer. Animal studies also show that inflammation can speed up the development of colorectal cancer. These findings show a strong link between inflammation and colorectal cancer [59, 60]. Indeed, KEGG pathway analysis prioritized the PI3K‐Akt signaling pathway secondly just below metabolic pathways, glorifying the firm interaction between growth factors, their receptors and the immune‐related genes including cytokines, their receptors and some mutual transcriptional factors as shown in Figure 9.

In this work, the most important hub gene, SRC, has been shown to be a new shared marker between CD and IBS. Targeting SRC could represent a promising strategy for developing new treatments for both conditions. It is, therefore, subjected to virtual screening via DrugRep server to discover potential repurposable drugs against this mutual gene, which, when blocked, can brake the progression of CD as well as IBS. Temoporfin, rimegepant, and eltrombopag, the top three lead candidates, upon docking gave a binding affinity of −11.1, 10.9, and −10.8 kcal/mol, respectively. This indicates the significantly strong binding to SRC and suggests their superior repurposable role. They formed different, yet multiple binding forces including H‐bonds, electrostatic attraction, and hydrophobics with the catalytic triad of the SRC active site (Figure 9). Basically, temoporfin is a photosensitizing agent that is widely used as a therapeutic option for oral cancers and periodontal diseases [61]. Rimegepant, on the flip side, is an antagonist of calcitonin gene‐related peptide receptor that has been approved to treat adults suffering from migraine with/without aura. It can also be used prophylactically for episodic migraine [62]. Prescribed for adults, the use of eltrombopag has been proven to reduce the incidence, the events of bleeding and, hence, permit an improved global platelet homeostasis and response. Patients with a platelet count < 20,000/mm^3^ are the most beneficiary [63]. Overall, these are the most promising FDA‐approved drugs that greatly inhibit SRC and, thus, their experimental validation is of particular interest and highly advisable since CD as well as IBS patients would benefit from this dual targeting in clinical practice. The experimental validation alone is sufficient to prescribe these therapeutics given the fact that they have already gained FDA approval for their efficacy and acceptable safety [64–66].

### 4.1. Study Limitations

Upon conducting this study, we noticed some of the potential limitations. These involve the combination of two different experimental types, that is, whether RNA‐seq or microarray may result in distinct outputs. Moreover, GSE164883 microarray data focused mainly on adolescents, which could be considered a confounding factor. Another critical aspect of the shortcomings came from the sample type taken from participants of dataset GSE63379, which is WBC compared with colon or intestinal biopsies of the other datasets. This might create a fundamental inherent differential expression. Lastly, this study is computational, and its consistency with experimental validation may not be identical.

## 5. Conclusions

This study revealed the molecular genetic similarities and differences between CD and IBS, two disorders that affect the GI tract and have common features and symptoms. By using an in silico systems biology approach, the study integrated and analyzed the RNA‐seq and microarray data of both diseases and identified 439 shared genes that were enriched for various cellular compartments, biological processes, and molecular functions. The study also discovered the transcriptional factors and miRNAs that regulate these genes and the protein interactions that mediate their functions. Among the shared genes, the study found that SRC was the most significant hub gene, which plays a key role in cell proliferation, metabolism, and apoptosis. The study also performed a virtual screening of approved drugs against SRC and suggested three potential drug candidates that can inhibit SRC activity and may have therapeutic benefits for both CD and IBS patients. These drugs are temoporfin, rimegepant, and eltrombopag, which have high binding affinity to SRC and low toxicity. The study recommended further experimental validation of these drugs in vitro and in vivo. This study provided novel insights into the molecular genetic mechanisms of CD and IBS and suggested new therapeutic avenues for these disorders based on SRC inhibition.

## Consent

The author has nothing to report.

## Conflicts of Interest

The author declares no conflicts of interest.

## Author Contributions

H.A‐M.: conceptualization, investigation, methodology, data curation, software, resources analysis, validation, writing—original draft, and writing—review and editing.

## Funding

No funding was received for this manuscript.

## Data Availability

The gene expression omnibus data (GSE146190, GSE164883, GSE166869, and GSE63379) are included within the article.
